# An Oxymetazoline-Based Hemostatic Solution Used with MTA for Pulpal Therapy: A Retrospective Study

**DOI:** 10.3390/children13010028

**Published:** 2025-12-24

**Authors:** Robert S. Jones, Hannah Lee, Jingqi Jia, Elise W. Sarvas

**Affiliations:** 1 Division of Pediatric Dentistry, Department of Developmental & Surgical Sciences, School of Dentistry, University of Minnesota, Minneapolis, MN 55455, USA; 2 Gunderson Health System, La Cross, WI 54601, USA; 3 Department of Dental Specialties, Mayo Clinic, Rochester, MN 55905, USA; sarvas.elise@mayo.edu

**Keywords:** vital pulp therapy, pulpotomy, primary teeth, ferric sulfate, oxymetazoline, adrenergic alpha-agonists, zinc oxide-eugenol, mineral trioxide aggregate

## Abstract

**Highlights:**

**What are the main findings?**

- MTA-based pulpotomies pretreated with a hemostatic solution of 0.05% oxymetazoline were found to be more successful in preventing radiographic pathosis compared to 20% ferric sulfate (FS)-based pulpotomies.
- Younger children had lower chances of developing radiographic and clinical signs of pathosis compared to older children.

**What is the implication of the main finding?**

- Oxymetazoline controls pulpal bleeding prior to MTA placement in a primary tooth pulpotomy, resulting in a relatively high chance of survival compared to FS after 36 months.
- Pulpotomy success depends on both age and the materials used.

**Abstract:**

**Background/Objectives:** The purpose of this study is to assess whether the addition of an oxymetazoline (OXY) hemostatic solution, which can be used to manage pulpal bleeding, maintains higher MTA survivability than pulpotomies treated with FS. **Methods**: In this retrospective cross-sectional study, patient data (*n* = 75) were used to assess radiographic and clinical signs and symptoms of pathosis in primary molars treated with a pulpotomy and a stainless-steel crown. Pulpotomies treated with FS (Group 1) were compared to those treated with MTA with OXY-induced hemostasis (Group 2). Restricted mean survival times (RMSTs) were calculated for the two groups, and Cox proportional hazards regression was used to analyze the effects of patient and practice level covariates on radiographic and clinical pathosis. **Results**: Cox proportional-hazard regression identified three potential covariates (age, pulpotomy groups, and procedure location) that predicted radiographic pathosis. The adjusted hazard ratio for Group 2 was 0.30 (95% CI: 0.11–0.82), indicating improved radiographic outcomes compared with Group 1 (*p* = 0.02). The 36-month RMST for Group 2 was 30.1 months (95% CI: 26.5–33.7) compared to 24.7 months (21.6–27.8) for Group 1 (*p* = 0.025). **Conclusions**: A pulpotomy utilizing OXY hemostasis prior to MTA placement led to a higher chance of pulpotomy survival than FS.

## 1. Introduction

The protocol for a primary tooth pulpotomy typically involves accessing the pulp canal, removing the coronal pulp tissue, and achieving hemostasis through direct pressure [1]. Residual bleeding may compromise the setting of pulp capping materials including mineral trioxide aggregate (MTA) and zinc oxide eugenol (ZOE) material. In many cases, pressure alone can achieve adequate hemostasis. However, achieving full hemostasis can take up to 5 min [2]. Furthermore, prolonged bleeding is a poor indicator of pro-inflammatory mediators [2]. For this reason, after primary pressure hemostasis is attempted via pressure, a pulpal hemostatic adjunct may be used to achieve secondary hemostasis and manage partially controlled bleeding [3].

Dental pulp tissue is innervated mainly by the autonomic nervous system, and activation of alpha-1-receptors creates vasoconstriction [4]. Oxymetazoline can induce hemostasis by activating alpha-1 receptors on vascular smooth muscles cells in the dental pulp of primary and permanent teeth [4,5,6]. Decongestant nasal sprays containing 0.05% oxymetazoline (OXY) applied directly on pulp tissue can manage bleeding during primary tooth pulpotomies [7]. OXY is commonly used in otolaryngology surgical procedures to manage bleeding by inducing vasoconstriction [8,9,10]. Its effect is transient and is eventually systemically absorbed via blood circulation. OXY is also used by anesthesia teams to manage bleeding during nasal intubation and is available in most hospital or outpatient surgical settings where dentists may perform dental rehabilitation under anesthesia [11].

Previous studies have demonstrated that primary tooth pulpotomies treated with MTA have better long-term survival than those treated with ferric sulfate (FS) [12]. After 24 months, MTA-treated pulpotomies have fewer signs of radiographic and clinical pathosis than those treated with FS [12]. The pulpal medicament FS, which can act as both a hemostatic agent and pulpal medicament covering the pulp, has been associated with long-term complications such as internal resorption [13]. There is currently a lack of clinical examination into the use of OXY as a hemostatic adjunct in a pulpotomy procedure with MTA. It is not known if OXY reduces the success of an MTA pulpotomy compared to FS. The main research question in this study is whether MTA pulpotomies pretreated with OXY are still more successful than FS pulpotomies.

In this study, a retrospective analysis examined the outcomes of primary molar pulpotomies with OXY used for hemostasis prior to MTA placement and compared survival to teeth treated with FS. The study also aimed to determine patient-associated and practice-related covariates that predicted radiographic and clinical pathosis. This approach compared OXY-MTA versus FS pulpotomies within a multivariate regression model, controlling for other covariates that may influence the presence of pathosis.

## 2. Materials and Methods

### 2.1. Study Design

This retrospective cohort study received Institutional Review Board approval at the University of Minnesota to review records of patients who underwent a pulpotomy procedure from 2018 to 2024. Pulpotomy procedures were performed by pediatric dental residents under the direct supervision of pediatric dentists. The referral-based clinic serves a large population of pediatric patients covered by public insurance programs. Prior to operative care, legal guardians signed treatment consent and consent for retrospective review of clinic charts for research purposes. Dental records were housed within an electronic health record (EHR, axiUm Academic Dental Software, Henry Shien, Melville, NY, USA) managed by the University of Minnesota. After IRB approval, the investigators used the informatics consulting services (ICSs) available through the University of Minnesota Center for Translational Science Institute (CTSI) to extract procedure specific records. The EHR system used current dental terminology codes (CDT, American Dental Association).

### 2.2. Inclusion and Exclusion Criteria

Inclusion criteria included primary molars treated with a therapeutic pulpotomy (D3220) and restored with a stainless-steel crown (D2930) by pediatric dental residents. Pulpotomies treated with MTA (neoMTA (Power/Liquid; Putty NuSmile, Houston, TX, USA) and pretreated with a hemostatic adjunct of 0.05% oxymetazoline (nasal solution of Afrin Original, Bayer Healthcare, Berlin, Germany, and generic equivalent) were included. A second comparison group was included. Pulpotomies treated with ferric sulfate (ViscoStat™, ferric sulfate 20%, Ultradent, South Jordan, UT, USA) with additional application of a reinforced zinc oxide eugenol dental restorative material (IRM, Dentsply Sirona, Charlotte, NC, USA) were included. The time frame (January 2018 to October 2024) for treatment codes was chosen to represent a period when providers used different materials based on professional judgment and historic practices.

The exclusion criteria included anterior primary teeth with pulp treatment, posterior teeth restored with a multi-surface restoration instead of a full coverage crown, material combinations not examined in this study, and cases that did not identify the materials used during the therapeutic pulpotomy. After the initial data records were pulled from the EHR, records that did not include any follow-up appointments or routine diagnostic radiographs were excluded from analysis. Chart records with non-diagnostic follow-up radiographs were also excluded. Records with a documented report of spontaneous pain prior to treatment were also excluded from analysis. Dental charts were excluded from the analysis if hemostasis was recorded in the note as not being established. For records where multiple D3220 procedures were performed within the same patient record and all radiographs were diagnostic, one tooth per patient record was randomly selected (https://www.random.org/lists/, accessed on 2 December 2025).

### 2.3. Calibration and Assessment

Clinical records and radiographs were organized by non-blinded reviewers (HL#1/RJ#2). Radiographs were deemed diagnostic if approximately 3 mm or more of the furcation was visible. Other decisions on the diagnostic readability of radiographs were determined by a blinded reviewer (ES#3). HL#1/RJ#2 organized a calibration dataset consisting of both pre-operative (pre-op, *n* = 20) and post-operative (post-op, *n* = 20) radiographs for assessment. These cases were compiled from a collection of records from a previously IRB-approved study and those in our study that either did not have a follow-up appointment (pre-op) or cases that were excluded. The pre-op assessment was used to assess a tooth’s condition prior to pulp treatment, such as peri-radicular findings. The calibration dataset was given to the blinded reviewer, who entered pre-op assessments of deeply carious teeth and post-op assessments of teeth treated with a pulpotomy and SSC. The blinded reviewer received the main dataset 3 days later and completed the assessment within approximately 2 weeks. One day following the assessment of the main study, the blinded reviewer returned to the two calibration datasets (pre-op and post-op). Intra-examiner agreement measurements were calculated using the weighted Kappa coefficient for both the pre- and post-op calibration datasets [14].

### 2.4. Study Variables

Dental record data were assessed for inclusion/exclusion by the non-blinded reviewers who examined treatment notes, confirmed treatment charges, and examined and recorded the interval of radiographs for a specific tooth. Patient- and practice-level data were assessed from the dental records. Patient-level data included the following: age at time of procedure, gender, tooth number, molar position, associated jaw (maxillary/mandible), behavior score, clinical signs of pathosis, and patient reported pain. The behavior score compared uncooperative behavior (Frankl score 1,2) to cooperative behavior (Frankl 3,4, and cases under general anesthesia). Pain was sub-classified as pain to percussion, pain associated with eating, and spontaneous pain. Practice-level data included the following: procedure location (clinic/operating room), date of procedure, presence of excessive bleeding, materials used in the pulpotomy, and type of final restoration. Pulpotomy materials used in the procedure were organized into two groups:

1. Group 1—pulpotomy procedure with ferric sulfate.
2. Group 2—pulpotomy procedure with oxymetazoline and MTA.

Blinded radiograph assessments examined patient-level factors associated with pre-op condition of the tooth. This pre-op assessment included whether the radiograph was of diagnostic quality, interproximal decay passing apically below the CEJ, signs of lateral and apical root resorption, presence of a furcal radiolucency, and the furcal radiolucency’s association with the eruption of succedaneous teeth to determine if it was physiological (the tooth was near exfoliation) or pathological. Post-op assessment of radiographs included at least one follow-up radiograph of diagnostic quality. The blinded examiner did not know the group or the follow-up time of the post-op radiograph. In some cases, the patient had more than one follow-up appointment and a single radiograph from each appointment was assessed by the blinded reviewer in temporal sequence.

### 2.5. Statistical Analyses

Patient and practice level variables were compared between the two pulpotomy treatment groups (Groups 1 and 2) to determine if any variable was not consistent between treatment groups. Chi-squared statistic test compared dichotomous outcome variables. For continuous data (age and follow-up time), normality testing (D’Agostino–Pearson test) determined if ANOVA or Kruskal–Wallis testing was appropriate.

Radiographic and clinical survival following the therapeutic pulpotomy were recorded as a function of the duration of time following the pulpotomy procedure. Radiographic and clinical success was defined as the absence of signs and symptoms of pathosis:

- Radiographic pathosis—internal resorption without perforation, internal resorption with perforation, and/or furcation radiolucency.
- Clinical pathosis—pain during chewing, spontaneous pain, gingival abscess, parulis, and/or swelling.

The presence of pathosis was defined as failure. Failure was interval-censored because the exact time of the first occurrence was unknown and was identified at a specific recall appointment. Absence of failure was right censored since the teeth were successful within the study period.

Cox proportional hazards regression was used to analyze the effects of independent study variables on tooth survival. Preliminary analysis included all variables in the regression model to assess the unadjusted hazard ratios and 95% CI for each variable. Final study variables were selected using a backward stepwise approach. All variables were entered into the model. Variables with *p* > 0.15 were removed from the model to determine the covariates in the final fitted model. The chi-squared test was used to assess the overall final fit of the Cox regression model and the relationship between the study variables and time. The concordance index (Harrell’s C-index) and its 95% confidence interval (CI) were used to assess the overall predictability of the final fitted model [15]. For the study variables included in the final model, adjusted hazard ratios (HRs) and 95% CIs were calculated.

Kaplan–Meier (KM) curves were used to examine the unadjusted group comparison of tooth survival. The restricted mean survival time (RMST) at 36 months was used for group comparison. All statistical analyses were performed using MedCalc software (v23.1.7, Ostend, Belgium).

## 3. Results

### 3.1. Examining Variable Distribution Within Groups

A query of the electronic dental health records identified 1360 patients with at least one primary molar treated with a pulpotomy and stainless-steel crown during the study period. The flow chart of inclusion is detailed in the supplemental material (Supplementary Material Figure S1). As a referral-based clinic, over half of the dental records included children who did not return for a single follow-up appointment. Over one-third of the total cases involved children who had a follow-up without dental radiographs due to the child’s ability to cooperate, short follow-up time, or provider decision. Of the remaining charts, evaluation of chart notes and radiographs identified 75 patients during the study period who had at least one primary tooth treated with a therapeutic pulpotomy (D3220) using materials in group 1 (*n* = 41) or group 2 (*n* = 34). No statistical difference was found in the distribution of patient- and practice-level variables between groups 1 and 2 in age, molar position, behavior, and location of the pulpotomy procedure (clinic/hospital setting) (Table 1). Additionally, the distribution of follow-up time was not significantly different and the 25th and 75th percentiles were comparable. While chart notes explicitly stated the hemostatic solution, pulpal medicament, and pulp capping material used during the pulpotomy procedure, documentation was incomplete regarding the use and duration of pressure hemostasis used prior to the placement of OXY or FS. Groups 1 and 2 had documented pressure hemostasis in 34.1% (*n* = 14) and 29.4% (*n* = 10), respectively. This difference was not significant between groups 1 and 2 (*p* = 0.66). No difference was found in the pressure hemostasis documentation between the clinic and hospital location sites (*p* = 0.19). No systemic hemodynamic adverse events were reported in the chart notes.

### 3.2. Calibration and Radiographic Assessment

**Figure 1:**
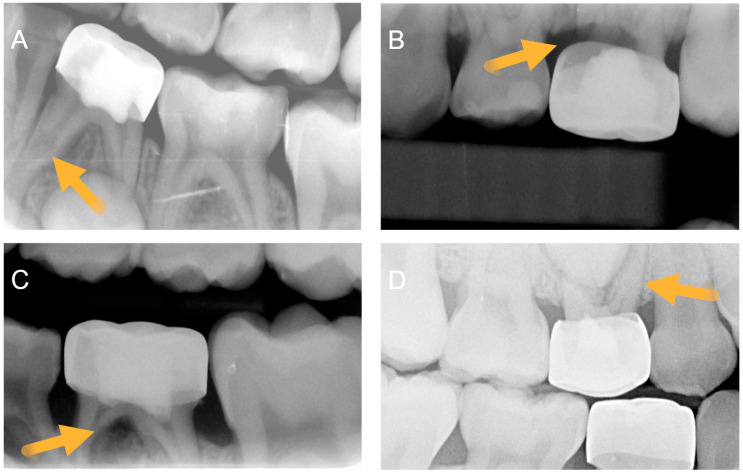
Post-pulpotomy radiographic assessment of (**A**) internal root resorption (IRR), (**B**) perforating IRR (IRR_P), and (**C**) furcal radiolucency (FRL). RR, IRR_P, and FRL were defined as signs of radiographic pathosis. (**D**) assessment of pulp canal calcification (PCC) that was defined as a non-pathological process. Arrows highlight representative conditions described.

Intra-examiner agreement measurements (weighted Kappa) coefficient for the pre-op calibration of caries extending below the CEJ was 0.69, and post-op calibration dataset for evaluating furcation radiolucency was 0.74. For the main post-pulpotomy assessment, the blinded reviewer assessed the presence of internal root resorption (IRR), perforating IRR (IRR_P), and furcal radiolucency (FRL) as radiographic signs of pathosis (Figure 1). After organizing the blinded review into the two treatment groups, all signs of pathosis were observed more frequently in Group 1 than in Group 2 (Table 2). A total of 20 teeth (48.8%) in Group 1 eventually developed at least one form of radiographic pathosis, with 4 having multiple pathoses. For Group 2, a total of 8 teeth (23.5%) developed one form of radiographic pathosis. Slightly over half of all treated teeth in both groups eventually developed signs of pulp canal calcification (PCC), which was assessed as a vital, non-pathological finding.

### 3.3. Survival Analysis

An initial radiographic analysis entered all variables into the Cox proportional hazards regression model and calculated the unadjusted hazard ratios (HRs) and 95% CI (Table 3). All variables were re-entered into the regression model and after a backward approach removed covariates whose significance was *p* > 0.15. This approach adjusted the HR of the final covariates in the regression model. For behavior, failure rates of pulpotomies in cooperative children were similar to those of uncooperative children (*p* = 0.36) and the variable was not used in the final regression model. The fourth column of Table 3 shows the adjusted hazard ratios and 95% CIs of three variables (age, pulpotomy material group, location) in the model after backward elimination. Age and pulpotomy group were the only statistically significant study covariates. Older age at the time of procedure was associated with 1.04 (HR) increase in radiographic pathosis per month of advancing age. Choice of Group 2, a pulpotomy with the use of OXY and MTA decreased the likelihood of radiographic pathosis (HR = 0.30) compared to Group 1. The chi-squared goodness of fit analysis determined a statistically significant relationship (chi-Squared = 8.32, *p* = 0.04) between time and variables in the model. The overall concordance index for the predictive variable was 0.66 (95% CI 0.56–0.76).

For clinical signs and symptoms of pathosis, study variables were reassessed and re-entered in a second Cox proportional hazards regression model (Supplementary Material-Table S1). The initial unadjusted hazard ratios (HRs) and 95% CIs for all variables were found to be insignificant. After backward elimination, age and group were retained (*p* < 0.15) in the regression model. Older age at the time of procedure was associated with increased clinical pathosis (*p* = 0.03). The choice of Group 2 was associated with an adjusted HR of 0.09 (95% CI 0.007–1.3) and was not statistically significant for preventing clinical pathosis (*p* = 0.08) compared with the choice of Group 1. The chi-squared goodness of fit analysis determined a statistically significant relationship (chi-Squared = 6.88, *p* = 0.03) between time and variables in the model. The overall concordance index for the predictive variable was 0.76 (95% CI 0.59–0.94).

**Figure 2:**
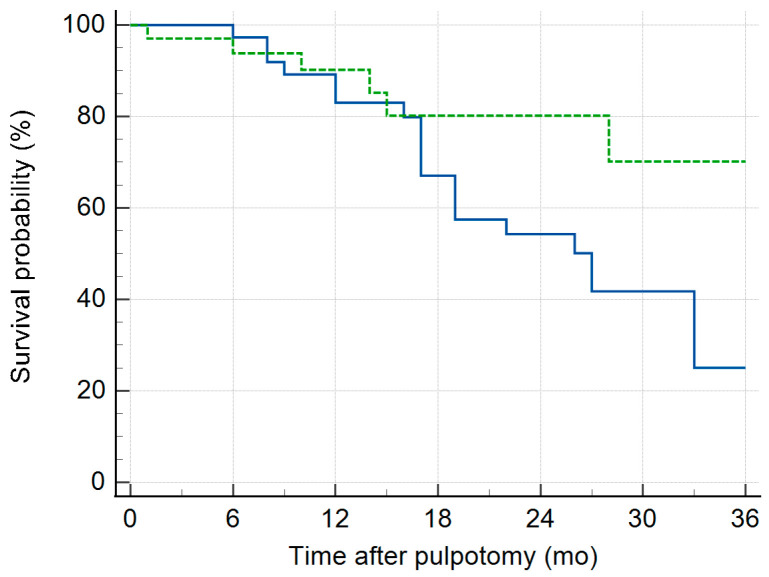
Radiographic survival curves from the Kaplan–Meier analysis over 36-month time period after pulpotomy treatment. Group 1 (solid line) and Group 2 (dotted). Radiographic evidence of pathosis (IRR, IRR_P, and FRL) indicate events where survival decreases.

**Figure 3:**
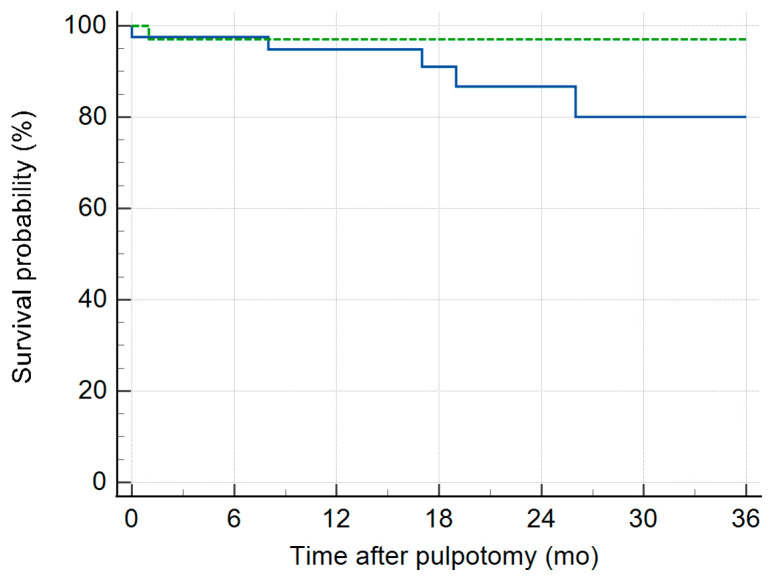
Clinical signs/symptoms curves from the Kaplan–Meier Analysis over 36-month time period after pulpotomy treatment. Group 1 (solid line) and Group 2 (dotted). Clinical signs/symptoms of pain, gingival abscess, parulis, and swelling at follow-up indicate events where survival decreases.

Kaplan–Meier (KM) survival curves illustrate the progressive decline in radiographic (Figure 2) and clinical (Figure 3) survival for both group 1 and 2 pulpotomies. Radiographic evidence of pathosis (IRR, IRR_P, and FRL) at follow-up appointments indicated events where survival decreased. Clinical signs/symptoms of pain, gingival abscess, parulis, and swelling at follow-up indicate events where survival decreased. Group 2 had a higher success than Group 1 on both the radiographic and clinical survival curves. The restricted mean survival time (RMST) was calculated as the area under the KM curve for both Group 1 and Group 2 up to the specified time point of 36 months (Table 4).

This 36-month RMST comparison allowed the two treatment groups to be assessed when the distribution of longest follow-up periods differed (Supplementary Material Figure S2). Group 2 had a statistically greater RMST for the absence of radiographic pathosis than Group 1. Group 2 had nearly 6-month greater probability of survival over a 36-month period compared to Group 1. For the presence of clinical signs and symptoms, no difference was found between the RMST for Groups 1 and 2.

## 4. Discussion

MTA-based pulpotomies pretreated with 0.05% OXY (Group 2) showed more radiographic and clinical success in this study compared to ferric sulfate (FS)-based pulpotomies (Group 1). These results are supported by past studies that have found MTA-based pulpotomies to be more successful than FS [12]. While MTA-based pulpotomies had higher success, it is noteworthy that both Group 1 and 2 had overall lower radiographic success than reported in some prospective trials [16]. This is an area for further investigation. One possible explanation is that both groups used a hemostatic agent as a substitute for cotton pressure hemostasis or used after brief pressure hemostasis. A limitation of the current study is that the documentation of the use of pressure hemostasis was very low.

Surprisingly, this study also found that younger age children had a lower chance of developing radiographic and clinical signs of pathosis compared to older children Older age has been found to be a determinant of pulpotomy success in other studies [16]. Older children were more likely to have radiographic pathosis. It has been speculated that younger pulps have more regenerative and less inflammatory potential.

Future investigations into MTA preceded by OXY pretreatment need to examine bleeding control with more precision. While many clinical trials may remove the confounder of excessive and residual bleeding for consideration into the study inclusion criteria [17,18,19], future studies should be designed in a manner that does not create overly ideal situations where bleeding control is established via cotton pressure over several minutes or cases are excluded if there is minor residual bleeding. Pragmatic trials are needed. An excessive bleeding determination that uses thresholds greater than 5 min is not practical when applied to scenarios of treating non-ideal behavior or in the operating room environment. Bleeding evaluation of 5 min can compound over many teeth needing treatment, and the use of such criteria is inappropriate for advanced behavior guidance techniques. Since residual bleeding may compromise the setting of MTA, which requires several hours before fully setting, OXY may improve outcomes in these case specific scenarios, but more investigation, specifically comparing MTA with and without the use of OXY, is needed. Furthermore, excessive bleeding may be caused by bacterial ingression into the canal space. In vitro data suggests that OXY, which contains quaternary ammonium compounds, has additional antimicrobial properties toward oral bacteria, and more clinically based studies are needed since the current study is limited to examining this direct bactericidal effect [3,20,21].

FS is often compared in meta-analyses directly with MTA as a two-group comparison. Notably, the three randomized clinical trials (RCTs) included in the most recent meta-analysis of vital pulp therapy that compared MTA and FS had an FS group in the comparison, which also included a pulp capping material of ZOE [12]. When used as a pulp capping material, ZOE, in the form of IRM, has methacrylate particles added to improve mechanical properties. This combination has also been shown to be substantially more cytotoxic on in vitro pulpal stem cells than MTA and Biodentine [22]. This may explain the high level of pathosis in the present study in the FS group.

There are several limitations to the study. The majority of pulpotomies performed in this referral-based practice had no follow-up appointment and were not included in the analysis. This may have influenced the results, especially outcomes based on behavior. Previous work has demonstrated that uncooperative behavior has been associated with poorer outcomes which was not found in the present analysis [23]. While providers encountered real world situations where the patient’s symptoms may not have been articulated due to behavior or the primary hemostasis was not ideal, there was a lack of standardization in the documentation of these scenarios in the evaluated clinical notes. Also, recall bias may have affected the evaluation of location. There was a non-significant trend that treatment in a clinic setting, compared to hospital setting, decreased the occurrence of a radiographic pathosis event. Since many GA patients are referred for treatment and do not return for routine care, patients treated in GA who did come back did so due to a problem, such as a clinically failed pulpotomy. Recall bias may have also affected the assessment of the influence of behavior on pulpotomy success if less cooperative patients did not return for follow-up. Given the referral nature of clinic cases, the analysis included a large proportion of cases with caries extending interproximally below the CEJ. This may also have affected the comparison, as the absence of caries interproximally below the CEJ was a factor that is related to improved outcome in a previous study [24].

OXY is a highly effective alpha-1-receptor agonist and can initiate hemostasis by vasoconstricting pulpal tissue. OXY is used extensively in otorhinolaryngology surgical procedures, suggesting biocompatibility and systemic safety [8,9,10,11,25]. The clinical protocol recommended using 1 drop (~0.05 mL) of 0.05% OXY-based nasal solution directly on pulp tissue for effective hemostasis [7], though exact volume was not recorded. The suggested volume is substantially less than the volume (1–2 mL) associated with adverse hemodynamic systemic effects of these over-the-counter nasal sprays in young children [26,27,28]. In the present study, no peri-operative adverse events were recorded.

Recent in vitro work has demonstrated that both FS and OXY have poor biocompatibility with human pulpal stem cells at full strength [21]. OXY becomes more biocompatible with dilutions, which likely happens when mixed with pulpal blood. At 1:10 dilution, OXY has near 80% biocompatibility compared to untreated controls [21]. Dilutions of FS, even at 1:10, were below 15% biocompatibility compared with controls [21]. This highlights the need to standardize OXY and FS application volumes and removal of excess medicament in future clinically based studies. Another alternative explanation for the lower success of MTA-based studies compared to ideal prospective study conditions is the reduced biocompatibility of MTA after OXY pretreatment. Future studies would benefit in comparing MTA with and without OXY.

The results of this work indicate that OXY used with MTA was associated with relatively high short-term success both radiographically and clinically. Future studies can also examine the effectiveness of OXY in other pulp therapy applications, such as direct pulp capping. With antimicrobial activity, nasal solutions with OXY may potentially be useful in cases of more advanced pulpitis in primary teeth.

## 5. Conclusions

OXY may be used as a hemostatic agent prior to MTA placement. In this retrospective study, this method led to higher pulpotomy survival than FS. Future prospective studies are needed to confirm this conclusion and examine the effect of OXY on MTA pulpotomy success.

## Figures and Tables

**Table 1:** Patient- and practice-related variables within treatment groups.

	Group 1 (*n* = 41)	Group 2 (*n* = 34)	*p*-Value
Age (months)	74.3	74.1	0.97 ^1^
Behavior (uncoop) ^2^	20.0%	8.8%	0.18 ^3^
IP caries below CEJ ^2^	70.0%	64.7%	0.63 ^3^
Gender (male)	46.3%	35.3%	0.34 ^3^
Jaw (mandible)	70.7%	55.9%	0.19 ^3^
Location (clinic)	92.7%	79.4%	0.09 ^3^
Molar position (2nd) ^4^	34.1%	47.1%	0.26 ^3^
Follow-up (months) ^5^	9-19-27	10-14.5-26	0.35 ^6^

^1^ mean with *p*-value from ANOVA analysis; ^2^ percentage of uncooperative behavior according to Frankl score assessment. Behavior and extensive interproximal (IP) decay passing below the cementoenamel junction (CEJ) is *n* = 40 (group 1) due to missing value/limitation in pre-op X-ray assessment; ^3^ from chi-square analysis; ^4^ percentage of second primary molars included in the treatment group; ^5^ interquartile range 25-50-75th percentile of the follow-up appointment in months per pathosis/survival event; ^6^ from Kruskal–Wallis test.

**Table 2:** Radiographic assessment of post-pulpotomy changes ^1^.

Group	IRR	IRR_P	FRL	PCC
1 (*n* = 41)	9 (22.5%) ^2^	4 (10.0%) ^2^	11 (26.8%)	22 (55.5%)
2 (*n* = 34)	2 (5.9%)	0 (0.0%)	6 (17.6%)	18 (52.9%)

^1^ internal root resorption (IRR); perforating IRR (IRR_P); furcal radiolucency (FRL); pulp canal calcification (PCC). Number of cases and percentage within the cohort are listed for each condition. For ^2^ A single case in Group 1, FRL confounded the assessment of IRR and IRR_P which changes total sample size to *n* = 40.

**Table 3:** Cox proportional-hazard regression models to assess variables related to radiographic signs of pathosis in post-pulpotomy assessment.

	HR (95% CI) ^1^	*p*-Value	Adjusted HR ^2^ (95% CI)	*p*-Value
Age (months)	1.03 (1.00–1.07)	0.07	1.04 (1.01–1.07)	0.02
Behavior (uncoop)	0.49 (0.11–2.26)	0.36		
IP caries below CEJ	2.18 (0.74–6.50)	0.16		
Gender (male) ^3^	0.77 (0.32–1.85)	0.56		
Group (2) ^4^	0.28 (0.10–0.76)	0.01	0.30 (0.11–0.82)	0.02
Jaw (mandible) ^5^	0.66 (0.29–1.53)	0.34		
Location (clinic) ^6^	0.38 (0.10–1.42)	0.15	0.31 (0.09–1.03)	0.06
Molar position (2nd) ^7^	1.52 (0.60–3.84)	0.38		

^1^ unadjusted hazard ratio using all variables. Age was a continuous variable. dichotomous HRs analysis relative to a (HR = 1.0) reference. ^2^ adjusted hazard ratio used a stepwise backward method of elimination of insignificant variables. ^3^ gender analysis of male with reference (1.0) female. ^4^ group 2 with reference (1.0) group 1. ^5^ mandible with reference (1.0) maxilla. ^6^ clinic location with reference (1.0) hospital location. ^7^ Second primary molar with reference (1.0) 1st primary molar.

**Table 4:** Restrictive mean survival time (months) (RMST) at time point of 36 months.

Group	Radiographic RMST (95% CI)	*p*-Value	Clinical RMST (95% CI)	*p*-Value
1	24.7 (21.6–27.8)	0.025	32.2 (29.6–34.8)	0.091
2	30.1 (26.5–33.7)		35.0 (33.2–36.8)	

## Data Availability

The data that supports the findings is available in the searchable Data Repository for University of Minnesota (DRUM), https://hdl.handle.net/11299/277439 (accessed on 12 December 2025). Based on the Repository’s policy, age and gender information were removed from the dataset and can be requested directly from the corresponding author.

## Supplementary Materials

The following supporting information can be downloaded at https://www.mdpi.com/article/10.3390/children13010028/s1, Figure S1: Flow Chart of Inclusion; Table S1: Clinical Cox proportional-hazard regression; Figure S2: Longest recall after pulpotomy.

## Abbreviations

The following abbreviations and codes are used in this manuscript.

ANOVA	Analysis of Variance
CEJ	Cementoenamel junction
FS	Ferric sulfate
FRL	Furcal radiolucency
Group 1	Pulpotomy procedure with ferric sulfate
Group 2	Pulpotomy procedure with oxymetazoline and MTA
HR	Hazard ratio
IRM	Intermediate restorative material
IRR	Internal root resorption
IRR_P	Perforating internal root resorption
KM	Kaplan–Meier
MTA	Mineral trioxide aggregate
OXY	Oxymetazoline
RSMT	Restrictive mean survival time
ZOE	Zinc oxide eugenol
