# Supplementary material for: An Oxymetazoline-Based Hemostatic Solution Used with MTA for Pulpal Therapy: A Retrospective Study
Source: Children (Basel). 2025 Dec 24;13(1):28. doi: 10.3390/children13010028 (PMC12839595; doi:10.3390/children13010028)
Supplement: Supplementary file 1 [file children-13-00028-s001.zip › children-4031698-supplementary.pdf]

children

Supplementary Materials

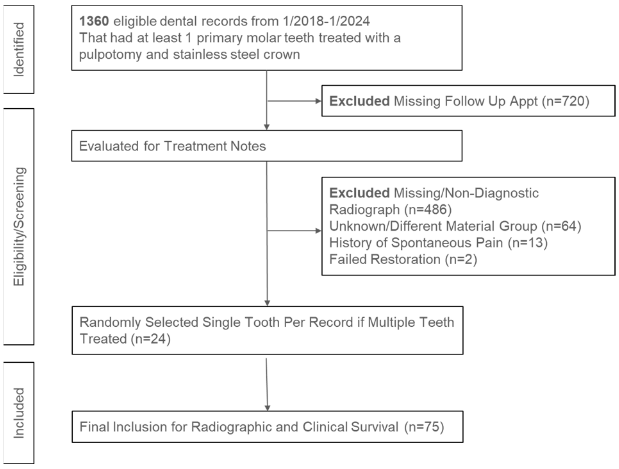

Figure S1. Flow Chart of Inclusion

Table S1. Cox proportional-hazard regression models to assess variables related to clinical signs and symptoms of pathosis in post-pulpotomy assessment. Variables below P <0.4 are presented.

	HR (95% CI)[1]	*P-value*	Adjusted HR[2] (95% CI)	*P*-value
Age (months)	1.05 (0.97-1.13)	0.18	1.08 (1.01-1.15)	0.03
Group (2)[3]	.078 (0.19-4.93)	0.052	0.09 (0.01-1.33)	0.08
Jaw (mandible)[4]	0.31 (0.06-1.65)	0.17		
Molar position (2nd)[5]	2.65 (0.38-18.74)	0.33		

[1]hazard ratio using all variables. age was a continuous variable. dichotomous HRs analysis relative to a (HR=1.0) reference

[2] adjusted hazard ratio used a stepwise backward method

[3] group 2 with reference (1.0) group 1

[4] mandible with reference (1.0) maxilla

[5]2nd primary molar with reference (1.0) 1st primary molar.

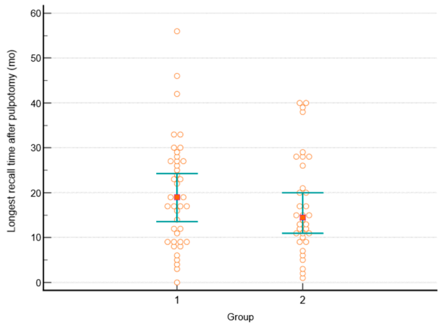

Figure S2. Longest recall time after pulpotomy in each material treatment group. Each individual case is plotted (circles) to the longest recall time in months. The distribution did not follow normal distribution. Medians and 95% confidence intervals of the median are presented. The difference between the groups was not statistically significant (P = 0.35) by Kruskal-Wallis testing.
